# *Pseudomonas putida* Fis Binds to the *lapF* Promoter *In Vitro* and Represses the Expression of LapF

**DOI:** 10.1371/journal.pone.0115901

**Published:** 2014-12-29

**Authors:** Andrio Lahesaare, Hanna Moor, Maia Kivisaar, Riho Teras

**Affiliations:** Institute of Molecular and Cell Biology, University of Tartu, Tartu, Estonia; Beijing Institute of Microbiology and Epidemiology, China

## Abstract

The biofilm matrix of the rhizospheric bacterium *Pseudomonas putida* consists mainly of a proteinaceous component. The two largest *P. putida* proteins, adhesins LapA and LapF, are involved in biofilm development but prevail in different developmental stages of the biofilm matrix. LapA is abundant in the initial stage of biofilm formation whereas LapF is found in the mature biofilm. Although the transcriptional regulation of the adhesins is not exhaustively studied, some factors that can be involved in their regulation have been described. For example, RpoS, the major stress response sigma factor, activates, and Fis represses LapF expression. This study focused on the LapF expression control by Fis. Indeed, using DNase I footprint analysis a Fis binding site Fis-F2 was located 150 bp upstream of the *lapF* gene coding sequence. The mapped 5′ end of the *lapF* mRNA localized the promoter to the same region, overlapping with the Fis binding site Fis-F2. Monitoring the *lapF* promoter activity by a β-galactosidase assay revealed that Fis overexpression causes a 4-fold decrease in the transcriptional activity. Furthermore, mutations that diminished Fis binding to the Fis-F2 site abolished the repression of the *lapF* promoter. Thus, these data suggest that Fis is involved in the biofilm regulation via repression of LapF expression.

## Introduction

The biofilm matrix consists of bacterial metabolites excreted from the cells into the medium. Depending on the environment and bacterial species, the central component of the matrix can be extracellular DNA, polysaccharides or proteins [1]. Contrary to the model organism *Pseudomonas aeruginosa*, which has a matrix known to be rich in polysaccharides [2], [3], the matrix of the rhizospheric bacterium *P. putida* consists mainly of proteins [4]. The two largest proteins in *P. putida*, the adhesins LapA and LapF, are known to be crucial for *P. putida*’s biofilm formation [5]–[8]. These adhesins are involved in biofilm development and prevail in different developmental stages of the biofilm matrix – LapA is necessary at the beginning of biofilm formation and LapF in mature biofilm [5], [7]–[10]. LapA is required for cell-surface interactions and therefore is needed for adhesion to plant roots [11] or seeds leading to biofilm initiation [5]. *P. putida*’s LapF provides cell-cell interactions, participating in the development of mature biofilm [10], [12]. *P. putida*’s LapF (PP0806) consists of 6310 amino acids and it has only one large repeat domain [5], [7], [12]. LapF is encoded by the first gene of the *lapFHIJ* operon. The other genes encode for an ABC transporter which could be involved in the secretion of LapF to the cell surface [12]. However, there is still no direct evidence that LapHIJ is involved in the transport of LapF [12].

LapF is extensively expressed in the stationary phase but not in logarithmically growing cells [8]–[10]. One reason for the *lapF* transcription activation in the stationary phase cells is a positive effect of RpoS, the sigma factor that is required for starvation and stress responses [10]. Additionally, several other indirect influences to the expression of LapF have been described. Martinez-Gil *et al.* (2014) monitored the expression of LapA and LapF by β-galactosidase assay and RT-PCR in several *P. putida* strains obtained by random mini-Tn*5* mutagenesis [9]. They found that the GacA/S two-component system is indirectly involved in the regulation of the LapF expression by modulating the expression of RpoS (sigmaS), and that c-di-GMP overproduction reduces LapF expression [9]. Furthemore, although the overexpression of the global regulator Fis enhances *P. putida* biofilm formation [13], it reduces the quantity of LapF about 4 times in the *P. putida* stationary-phase cells [8].

Fis is a small DNA-binding and -bending homodimeric protein, which is known as a trigger of fast growth in *Enterobacteriaceae* [14], [15]. It is speculated that Fis is substitutable by other nucleoid-associated proteins such as IHF, HU, H-NS or Dps in *Escherichia coli*, though none of the other proteins have noticeable amino acid sequence similarity to Fis [14]. However, although *E. coli fis* knock-out mutants are viable, it seems that the deletion of the *fis* gene is lethal for *Pseudomonas* species [13], [16], [17]. Fis participates in several important processes such as regulation of transcription and recombination [14], [15], but it is not commonly associated with biofilm development. Although the involvement of Fis in biofilm formation has been described in some studies [18]–[22], no one has shown the direct transcription regulation of adhesin genes in *P. putida* by Fis.

In this study, the 5′ end of the *lapF* mRNA is mapped, and the location of the *lapF* promoter is determined. A Fis binding site Fis-F2, which overlaps the *lapF* promoter, is identified by DNase I footprint and gel-shift assays. The adverse effect of Fis overexpression to the transcription from the *lapF* promoter is shown by a β-galactosidase assay. However, when testing a mutated Fis-binding site Fis-F2-mut, Fis does not protect the DNA against DNase I cleavage, nor does it repress transcription from *lapF* promoter, confirming that Fis directly represses the *lapF* promoter.

## Materials and Methods

### Bacterial strains, plasmids, oligonucleotides and media

The bacterial strains and plasmids used in this study are described in Table 1. Bacteria were grown in LB medium [23]. Solid media contained 1.5% Difco agar. Antibiotics were added at the following concentrations: ampicillin, 100 µg ml^−1^; gentamicin, 10 µg ml^−1^; kanamycin, 50 µg ml^−1^; penicillin, 1500 µg ml^−1^; streptomycin, 200 µg ml^−1^. *E. coli* was incubated at 37°C and *P. putida* at 30°C. Bacteria were electrotransformed as described by Sharma & Schimke [24]. *E. coli* strain DH5α (Invitrogen), was used for DNA cloning.

**Table 1:** Bacterial strains and plasmids used in this study.

Strain and plasmid	Genotype or description	Source/reference
***E. coli***		
DH5α	*supE44* Δ*lacU169*(f80 *lacZ*ΔM15) *recA1 endA1 hsdR17 thi-1 gyrA96 relA1*	Invitrogen
***P. putida***		
PSm	PaW85; chromosomal mini-Tn*7*-ΩSm1 (Sm^r^)	[13]
F15	PaW85; chromosomal mini-Tn*7*-ΩGm-term-*lacI* ^q^-P*_tac_*-*fis*-T1T2 (Gm^r^)	[13]
**Plasmids**		
pBBR1-MCS-5	Cloning vector (Gm^r^)	[27]
pBLK	1033 bp DNA fragment containing Km^r^ gene cloned into pMC5-lacZ opened with BglII and PaeI (Km^r^)	This study
pBLKT	Promoter probe vector, a 452 bp DNA fragment containing the T1T2 transcription terminator cloned into pBLK opened with BcuI and PdiI (Km^r^)	This study
pKST1T2	pBluescript KS(+) containing the transcription terminator sequence T1T2 (Amp^r^)	[29]
pHLU102	Promoterless *lacZ* gene	[26]
pMCS5-lacZ	3320 bp fragment containing the *lacZ* gene cloned into pBBR1-MCS-5 by PaeI and Acc65I restrictases (Gm^r^)	This study
pUTmini-Tn*5*Km2	Suicide vector, source of Km resistance gene (Amp^r^, Km^r^)	[41]
pBLKT-F-Fis	177-bp-long promoter region of the *lapF* gene cloned into pBLKT BamHI site (Km^r^)	This study
pBLKT-F-Fis-mut	177-bp-long promoter region of the *lapF* gene with the mutated Fis-F2 site cloned into pBLKT BamHI site (Km^r^)	This study
pLA1-12	Carrying LF2 site in left end DNA of Tn*4652*; (Amp^r^)	[30]
pRA1-12	Carrying RF1 site in right end DNA of Tn*4652*; (Amp^r^)	[30]

### Prediction of Fis-binding sites on the promoter region of the *lapF* gene

Possible Fis-binding sequences on the promoter regions of the *lap* genes were predicted using the *E. coli* Fis-binding sites matrix [25] and the matrix-scan program available at the Regulatory Sequence Analysis Tools homepage (http://rsat.ulb.ac.be/). The −500 bp to +100 bp DNA region of the *lapF* gene was used for the prediction of potential Fis-binding sites. The Markov model of zero order (Bernoulli model), organism-specific probability of nucleotides in the upstream region of genes in *P. putida* KT2440 and a *P*-value upper threshold of 0.001 were selected for the conditions of the background model. The rest of the parameters were left at the program’s default values.

### DNA manipulations

The promoter probe vector pBLKT (Table 1) was constructed to measure β-galactosidase activities. The 3320-bp DNA fragment containing the promoterless *lacZ* reporter gene from pHLU102 [26] was cloned into pBBR1-MCS-5 [27] using the PaeI and Acc65I restrictases, resulting in pMCS5-lacZ (Table 1). The Km^R^ gene was amplified by PCR from the plasmid pUTmini-Tn*5* Km2 [28] by KmSac primers (5′-CAGGAGCTCGTTCGATTTATTCAACAAAGCC-3′). The 1033-bp PCR fragment containing the Km^R^ gene was cleaved with Ecl136II and inserted into pMCS5-lacZ, which was opened with PaeI and BglII and thereafter blunted by Klenow I fragment, resulting in pBLK (Table 1). The pKST1T2 [29] was cleaved with BcuI and EcoRV and a 452-bp DNA fragment containing the T1T2 transcription terminator was obtained. The T1T2 fragment was inserted into pBLK, which was opened with PdiI and BcuI restrictases, resulting in the promoter probe vector pBLKT (Table 1).

Two pBLKT derivatives containing the *lapF* promoter region were constructed. The *lapF* promoter regions were amplified by PCR, and both fragments were cloned into the pBLKT BamHI site. A 235-bp PCR product was amplified by LapF-fw (5′-TAGATCTTTCGCTGAGGCTTTTCTAC-3) and PP0806-rev (5′-TGGATCCACTTCGGATTGCTTATCGG-3′) oligonucleotides. The PCR product was cut with BglII and BamHI and resulted in two fragments. The fragment containing the −198 bp to −22 bp region of the *lapF* upstream DNA was used to obtain pBLKT-F-Fis.

**Figure 1:**
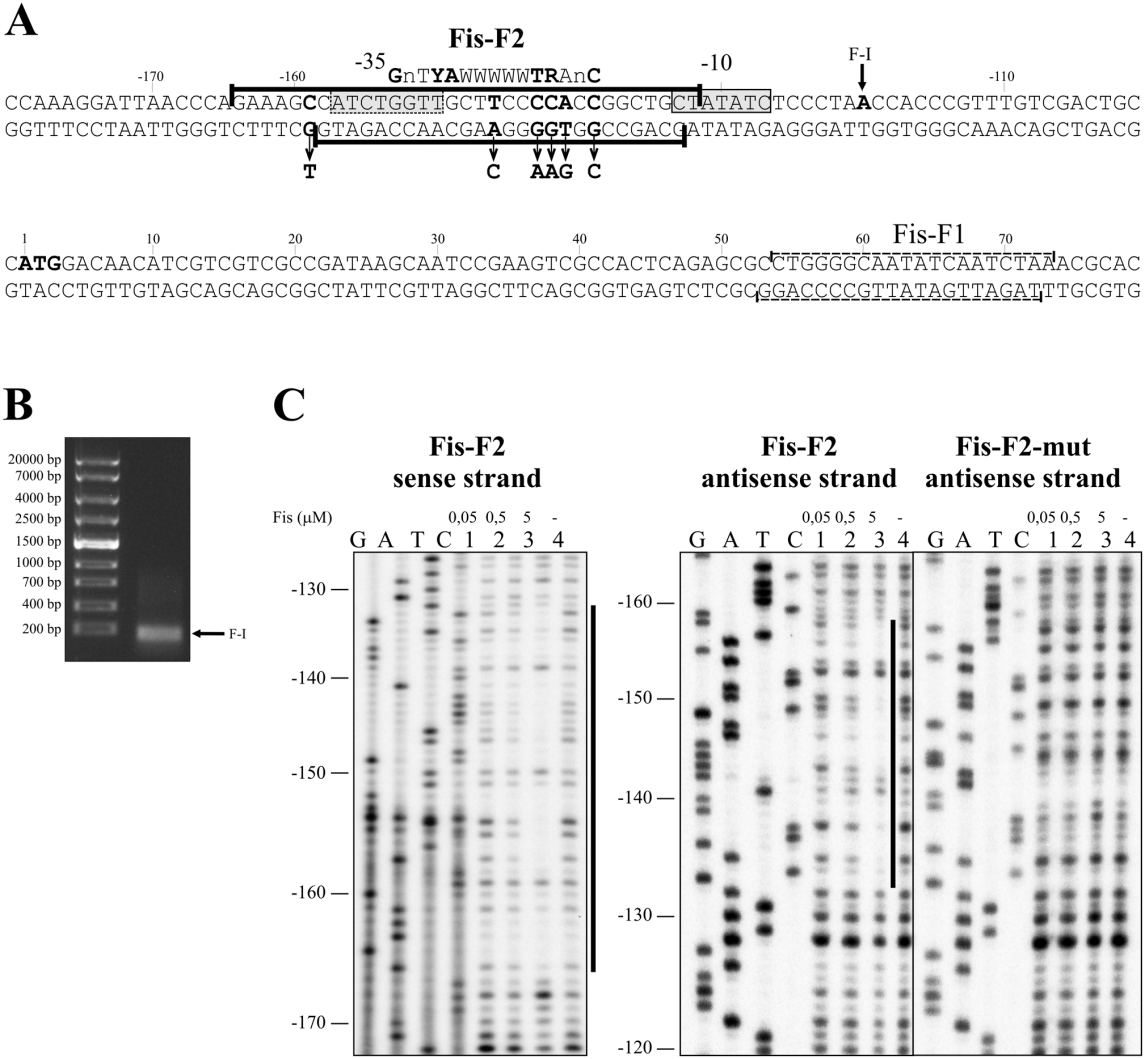
Mapping of Fis binding sites, promoter, and 5′ end of mRNA at the *lapF* promoter region. (A) Sequence of the *lapF* promoter region and downstream DNA. The start codon of the *lapF* gene is shown in bold. The first nucleotide of the mRNA 5′ end is written in bold and is designated as F-1. The potential –10 and –35 elements of the *lapF* promoter are shown in grey boxes. The Fis binding site Fis-F2 is shown in black brackets and the *in silico* predicted Fis binding site Fis-F1 is shown in dashed brackets. The *E. coli* Fis binding consensus according to Finkel and Johnson (1992) and Shao *et al.* (2008) is shown above the *lapF* promoter sequence [36], [37]. The most important nucleotides for the *E. coli* Fis binding are shown in bold. The point mutations in the Fis-F2 antisense strand are indicated by arrows (B) Agarose-gel electrophoresis of cDNA amplified by the RACE method for the identification of the *lapF* mRNA 5′ end. The arrow points to the PCR product used to determine the mRNA 5′ end. (C) Protection of the *lapF* upstream DNA against DNase I cleavage by binding of Fis on the sense and the antisense strands. Lines at the right side of the panels indicate the regions protected by Fis from DNase I cleavage at the positions −165 to −132 on the sense strand and –158 to −133 on the antisense strand corresponding to Fis-F2.

For the construction of pBLKT-F-Fis-mut, site-directed mutagenesis of wild-type Fis-F2 was performed using two sequential PCRs and the *P. putida* PSm chromosome as a template. These amplifications resulted in a fragment with five substituted nucleotides in the Fis-F2 binding site but otherwise identical to the one that was used for obtaining pBLKT-F-Fis. In the first PCR, the oligonucleotides LapFII-mut carrying five substitutions (5′-CATCTGGTTGCT**G**CC**TTC**C**G**GGCTGCTATATC-3′, **substitutions in bold) and PP0806-rev were used for DNA amplification (see sequence in Fig. 1A). In the second PCR, LapF-fw and the product of the first PCR were used as primers for DNA amplification from the *P. putida* PSm chromosome. Thereafter the PCR fragment was restricted with BamHI and BglII. The fragment containing the −198 bp to −22 bp region of the mutated *lapF* upstream DNA was inserted to pBLKT, resulting in pBLKT-F-Fis-mut. All designed plasmids were sequenced in order to exclude PCR-generated errors in the cloned DNA fragments.**

### DNase I footprinting

DNase I footprint assays were performed for the identification of *P. putida* Fis-binding sequences on the *lapF* promoter region. PCR-amplified fragments were used for DNase I footprint assay and were generated as follows. The 149 bp-long DNA fragment upstream of the *lapF* gene was amplified using the LapF-fw and LapF-down2 (5′-CGTAACAGAGCCCTTTCAG-3′) oligonucleotides to identify Fis binding sites upstream of the *lapF* gene. Depending on the template (pBLKT-F-Fis or pBLKT-F-Fis-mut), the PCR-amplified fragments contained either the wild-type Fis-F2 or the mutated Fis-F2-mut Fis-binding site. The following procedures: labelling PCR products with [γ-^32^P]-ATP, preparing reaction mixtures and gel electrophoresis were carried on as described in Teras *et. al* (2009) [16].

### Gel mobility shift assay

The same labelled PCR-amplified fragments that were used for DNase I footprint assays, were used for the gel mobility assay. Additionally, the non-labelled PCR product containing the Fis binding site LF2 [30] and a PCR product without Fis-binding site RF1 [30] were used in out-competition experiments. The unlabelled DNA fragment LF2 was amplified using the oligonucleotides TnLsisse (5′-GCAAAGACTGCTTCGCGCCC-3′) and SIDD-2 (5′-AGAGCTCCTGTACGTGCGCTT-3′). Oligonucleotides PRH8 (5′-GCTGAGCTCAGACGGTGGATGACCAGC-3′) and Tnots (5′-GGGGTTATGCCGAGATAAGGC-3′) were used for the amplification of unlabelled DNA RF1. Plasmids pLA1-12 and pRA1-12 [30] were used for amplifying LF2 and RF1, respectively. Amounts of competing DNA in the reaction mixes were calculated in molecules.

Binding reactions with purified *P. putida* His-tagged Fis were carried out with 2×10^10^ molecules (750–1000 c.p.m.) of labelled DNA fragment in a reaction buffer (24 mM Tris/HCl pH 7.5, 50 mM KCl, 10 mM MgCl_2_, 1 mM CaCl_2_, 0.1 mM EDTA, 5% glycerol, 0.05 µg BSA µl^−1^ and 0.05 µg salmon sperm DNA µl^−1^) in a final volume of 20 µl. The mixtures were preincubated for 20 min at room temperature. After incubation, reaction mixtures were applied to a 5% non-denaturing polyacrylamide gel buffered with TBE (50 mM Tris, 60 mM boric acid, 5 mM EDTA; pH 7.5). Electrophoresis was carried out at 4°C at 10 V cm^−1^ for 3 h. Gels were vacuum dried and exposed to a Typhoon Trio screen (GE Healthcare).

### Identification of 5′ ends of mRNA by RACE

The mRNA 5′ end of the *lapF* gene was identified by RACE (rapid amplification of cDNA ends) as described by Sambrook and Russell (2001) [31]. 1.5 µg of purified total RNA and the LapF-RACE1 primer (5′-GCCGACGAAGACCATATC-3′) were used for the amplification of the first strand of cDNA. The second strands of cDNA were amplified using primers Adapt-pikkC (5′-GACTCGAGTCGACATCGA(C)_17_-3′) or Adapt-pikkT (5′-GACTCGAGTCGACATCGA(T)_17_-3′), with 5′ ends binding accordingly to poly-G or poly-A, synthesised by terminal deoxynucleotidyltransferase (TdT) to the 3′ ends of the first strand of cDNA. For the second PCR, the Adapt-lyh (5′-GACTCGAGTCGACATCG-3′) and PP0806-rev primers were used. Zymo Research DNA Clean & Concentrator-5 kit was used for DNA purification between the RACE stages.

### Measurement of β-galactosidase activity

To measure β-galactosidase activities, *P. putida* cells were grown in LB medium with or without 1****mM IPTG supplementation for 18 hours. As a source of β-galactosidase, the pBLKT-F-Fis and pBLKT-F-Fis-mut constructs containing *lapF* promoter region in front of the *lacZ* gene were used (Table 1). The measurement of β-galactosidase from cell suspension was performed according to the protocol of Miller (1992) [23]. At least eight independent measurements were performed.

### Statistical analysis

The factorial analysis of variance (ANOVA) method and *post-hoc* Bonferroni test at a significance level 0.05 were used to assess the variability of experimental data. The calculations were performed using Statistica 10 software.

## Results and Discussion

### Mapping of the Fis-binding sites in the *lapF* promoter region

We have previously shown that Fis overexpression decreases the quantity of LapF about four times in *P. putida* cells [8]. Therefore, we were interested in the possible interaction of Fis and the *lapF* promoter region. We used *in silico* prediction of Fis-binding sites on the upstream region of the *lapF* gene to obtain initial information for later transcriptional studies. Surprisingly, no Fis-binding sites were predicted *in silico* for the upstream sequence of *lapF* under the selected conditions (detailed information in the Material and Methods section). However, there was one predicted Fis-binding sequence (Fis-F1) approximately 65 bp downstream the *lapF* start codon (Fig. 1A). The weight-score and p-value for Fis-F1 were respectively 6.5 and 0.0002 for the sense strand and 5.0 and 0.00091 for the antisense strand. The applied matrix’s maximum weight score was 12.5 and minimum weight score was −28. Despite the *in silico* prediction, we could not verify Fis binding to the predicted Fis-F1 sequence by DNase I footprint analysis (data not shown). Instead, DNase I footprint analysis revealed another Fis-binding site, Fis-F2, located approximately −150 bp upstream of the *lapF* gene (Fig. 1A and C).

To identify the promoter sequences of the *lapF* gene, we mapped the 5′ end of the *lapF* mRNA by RACE method. We identified one 5′ end of the mRNA at the position −120 upstream of the *lapF* coding sequence (Fig. 1A and B). Since the *lapF* transcription has been previously shown to be activated by RpoS in stationary phase cells [10], we expected to find a recognisable –10 element of the sigmaS-dependent promoter. It is known that sigmaS-dependent promoters, like the *Pm* promoter from the *P. putida* TOL plasmid and the *PalkS* promoter from *Pseudomonas oleovorans* OCT plasmid, depend on RpoS both in *E. coli* and in *P. putida* [32]–[34]. Therefore we used the consensuses of *E. coli* sigmaS-dependent promoters to predict *lapF* promoter elements. A putative –10 promoter element sequence GCTATATC was located six nucleotides upstream of the mapped 5′ mRNA end F-I (Fig. 1A). This is similar to the –10 consensus sequence of the *E. coli* sigmaS-dependent promoters KCTAYACT, where “K” is G or T and “Y” is pyrimidine [35]. The sequence of the –35 promoter element is not well-conserved among *E. coli* sigmaS-dependent promoters and can differ from the consensus of the sigma70-dependent –35 element TTGACA [35]. Even more, the preferred spacer length between the –10 and –35 elements is more flexible compared to sigma70-dependent promoters, varying from 15 to 19 nucleotides [35]. However, although the sequence of the –35 element is not conserved, modifications in this region can change the transcription initiation from sigmaS-dependent promoters [35]. Indeed, no recognisable sequence was found (Fig. 1A) that would be similar with the –35 element of sigma70-dependent promoters’ consensus TTGACA [35]. Considering the overlapping position of the –10 hexamer and the Fis binding site Fis-F2 it was plausible that Fis could repress transcription from the *lapF* promoter by impeding RNA polymerase binding.

To confirm direct binding of Fis to the promoter of the *lapF* gene, we mutated the five nucleotides of Fis-F2 that could be the most important for Fis binding (Fig. 1A). According to Finkel and Johnson (1992) and Shao *et al.* (2008) the most important nucleotides for *E. coli* Fis binding are the 1^st^, 4^th^, 5^th^, 11^th^, 12^th^ and 15^th^ nucleotides in the GnTYAWWWWWTRAnC consensus. In the consensus, “Y” is pyrimidine, “W” is A or T, “R” is purine and “n” is any nucleotide [36], [37]. Considering the fact that the potential –35 hexamer of the *lapF* promoter is located within the Fis-F2 binding site, we could only mutate the nucleotides that did not belong to the potential –35 and –10 elements of the promoter (Fig. 1A and C). The C nucleotide substitution with A at the position −159 bp from the *lapF* gene happened by chance during PCR. However, this mutation was inside the Fis-F2 site without overlapping the potential –35 element. Therefore, we decided to use this construct for following experiments.

The DNase I footprint analysis carried out with the Fis-F2-mut DNA and the purified Fis revealed that unlike the wild-type Fis-F2, Fis did not prevent DNase I cleavage of the Fis-F2-mut sequence (Fig. 1C). Thus, Fis did not bind the mutated Fis-F2 sequence or bound it with a weak affinity, which was undetectable by the DNase I footprint analysis.

**Figure 2:**
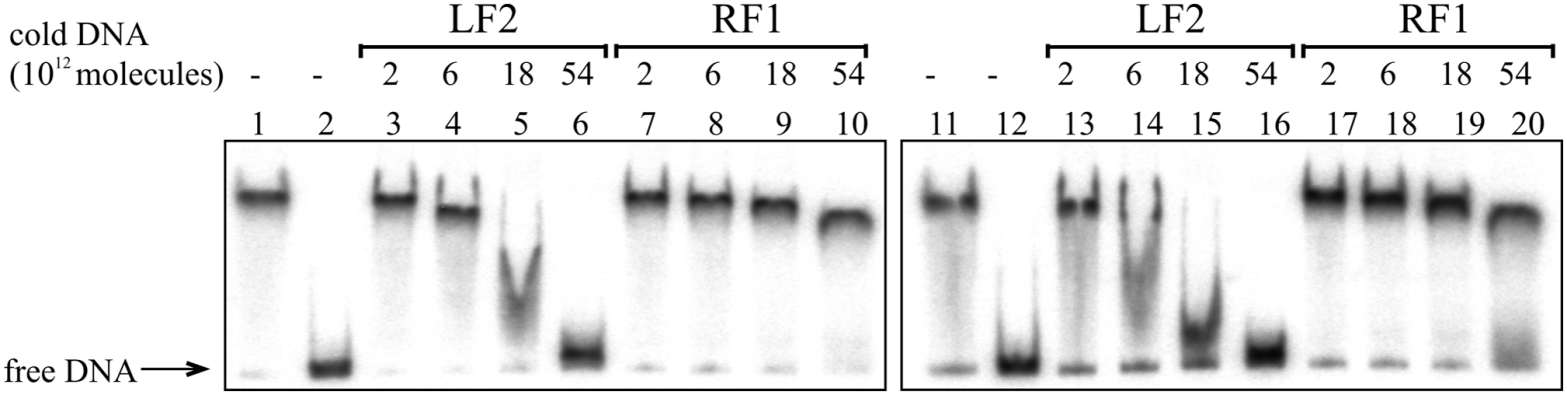
Gel shift assay of the Fis binding to the *lapF* promoter DNA containing the wild-type Fis binding site Fis-F2 and the mutated site Fis-F2-mut. 2×10^10^ molecules of radioactively labelled PCR products containing Fis-F2 (lanes 1–10) and Fis-F2-mut site (lanes 11–20) were used for Fis binding. Fis was outcompeted from Fis-DNA complex with unlabelled PCR product containing the Fis binding site (LF2) and PCR product without Fis-binding site (RF1). Added unlabelled DNA was calculated in molecules. 0.46 µM Fis was used in each reaction mixture except mixtures without Fis in lanes 2 and 12.

Additionally, Fis binding to the DNA containing Fis-F2 was assessed by gel mobility shift analysis. To assess Fis specific binding to the Fis-F2 sequence, unlabelled DNA containing the Fis-binding site LF2 from the left end of Tn*4652* [30] was used to outcompete Fis from the *lapF* promoter DNA-Fis complex (Fig. 2). Although Fis bound relatively similarly to the Fis-F2 and Fis-F2-mut DNA fragments, LF2 outcompeted Fis from Fis-F2-mut complex more easily than from Fis-F2 complex (Fig. 2). Thus, the introduced mutations in the Fis-F2 sequence had an adverse influence on Fis binding *in vitro*.

### The impact of Fis on the transcription of the *lapF* gene

To elucidate the possible effect of Fis on the transcription of the *lapF* gene, the promoter region was cloned to the front of the reporter gene *lacZ* in a promoter probe vector pBLKT (Table 1). The β-galactosidase activity was measured in stationary-phase cells of the *P. putida* strains PSm (wild-type) and F15 (IPTG-inducible Fis overexpression strain). Indeed, we could solely examine Fis’s impact to *lapF* expression using the *fis* overexpression strain F15, since all our attempts to reduce Fis’ amount by *fis* gene deletion or conditional gene expression resulted in a lethal genotype or unexpected recombination of the native *fis* to somewhere in the *P. putida* genome [13], [16].

**Figure 3:**
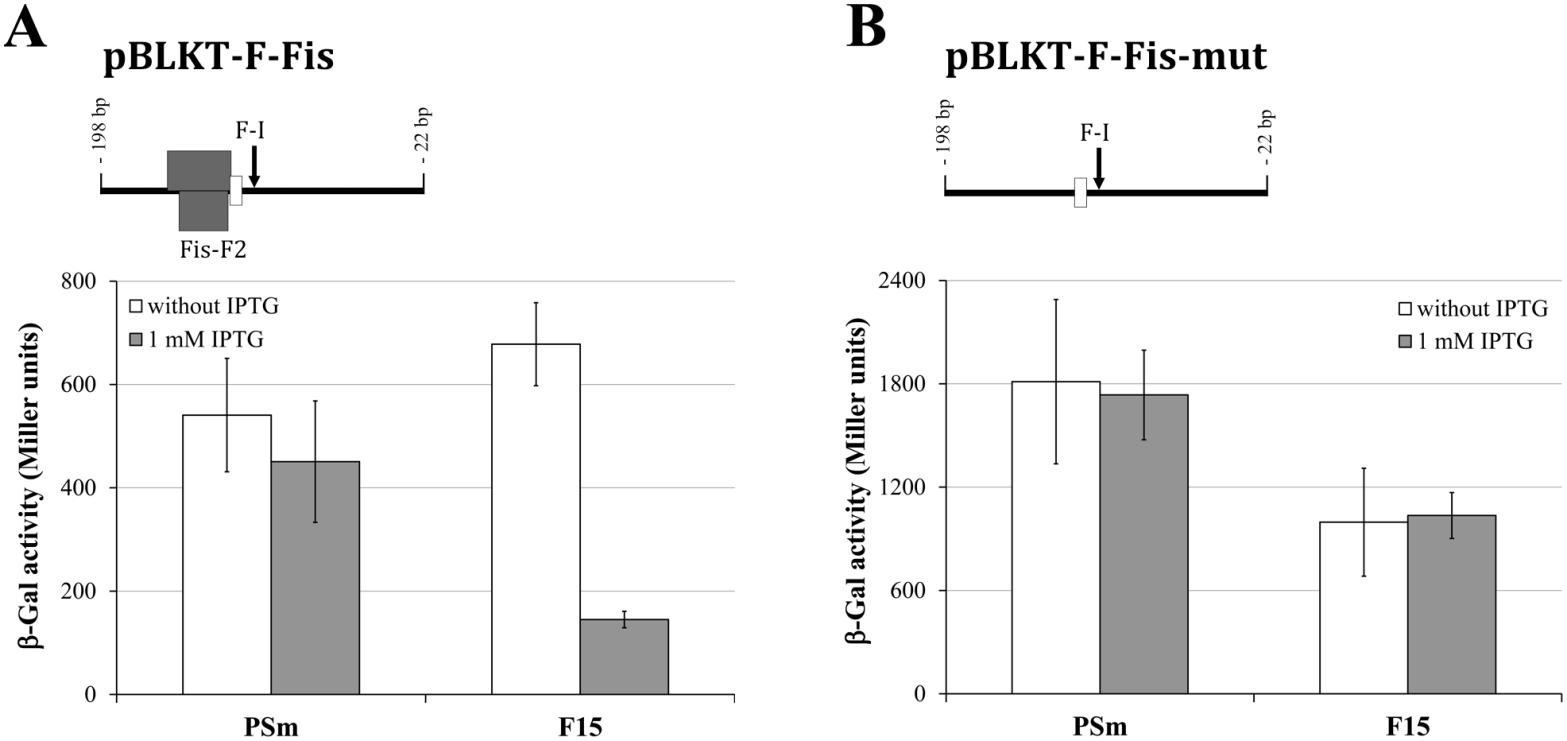
Effect of overexpression of Fis on the level of transcription from the *lapF* promoter. β-Galactosidase (β-Gal) activity expressed from the *lapF* promoter-lacZ reporter constructs were measured in the wild-type strain PSm and the Fis-overexpressing strain F15 of *P. putida* grown for 18 hours in LB medium supplemented with 1 mM IPTG and without IPTG supplementation. Vertical bars denote 95% confidence intervals of means. Data of at least 8 independent measurements is shown. The length of the *lapF* promoter region inserted upstream of the *lacZ* reporter gene in different pBLKT constructs is shown above diagrams. The Fis-binding site Fis-F2 (gray box) and 5′ end of *lapF* mRNA F-I (arrow) are shown in drawings.

The influence of IPTG to the *lacZ* gene expression was assessed in LB medium. Comparing the LacZ activities of the PSm cells carrying the pBLKT derivates, we did not observe any statistically significant differences in the results when the cells were grown in LB medium with and without 1 mM IPTG (Fig. 3). This control experiment demonstrated that IPTG itself has no influence on the expression of the *lacZ* gene in *P. putida*. Also, we measured the expression of *lacZ* from the promoterless pBLKT in the cells of PSm and F15. In all cases, the expression of the *lacZ* gene resulted in β-galactosidase activities lower than 0.9 Miller Units and no effect of the presence of IPTG in the LB medium or the use of the host strain was observed (data not shown).

The LacZ activity was measured in *P. putida* harbouring the pBLKT constructs containing the *lapF* promoter region in front of the reporter gene *lacZ*. As expected, Fis overexpression drastically reduced the β-galactosidase activity in cells carrying the *lapF* promoter-*lacZ* transcriptional fusion. We measured LacZ activity in *P. putida* harbouring pBLKT-F-Fis, the plasmid that contained the DNA region encompassing −198 to −22 bp from the *lapF* start codon inserted in front of the reporter gene *lacZ*. The LacZ activity was comparable in the wild-type cells (PSm) and F15 grown without IPTG and did not have statistically significant differences. However, Fis overexpression in F15 reduced the LacZ activity 4.2 times (*p*<0.001) compared to F15 grown without IPTG (Fig. 3A). Thus, the β-galactosidase results confirmed our previous finding that Fis overexpression decreases the amount of LapF in *P. putida* stationary-phase cells when LapF is efficiently expressed [8].

We employed a pBLKT-F-Fis-mut construct to elucidate Fis’s effect on the *lapF* gene’s transcription by β-galactosidase assay. The pBLKT-F-Fis-mut construct was identical to pBLKT-F-Fis, except for point mutations in the Fis-F2 binding site that reduced Fis binding in *in vitro* assays. Fis overexpression did not decrease the activity of LacZ in *P. putida* F15 harbouring pBLKT-F-Fis-mut (Fig. 3B), indicating direct regulation of LapF expression by Fis. However, if compared to the wild-type strain PSm, the LacZ activity in F15 remained 1.8 times (*p*<0.001) or 1.7 times lower (*p* = 0.001), depending on the absence or presence of 1 mM IPTG in the growth medium. In addition, the LacZ activity measured in the wild-type strain PSm cells carrying the pBLKT-F-Fis-mut construct was approximately three times higher than that in cells with pBLKT-F-Fis. Hence, we suppose that the introduced mutations in the Fis-F2 site increased the general expression of LacZ, but at the same time had an adverse effect on Fis binding.

Thus, the Fis-binding site Fis-F2 is needed for Fis to repress the *lapF* promoter. Both the results of the β-galactosidase assay (Fig. 3) and the DNase I footprint analysis (Fig. 1) indicate that the transcription of *lapF* is repressed by Fis. Moreover, our data confirm our previous finding that Fis overexpression can diminish the quantity of LapF in *P. putida* stationary phase cells, where LapF is strongly expressed [8].

Considering the dependence of RpoS and Fis amounts on *P. putida* growth phases, the expression of LapF seems to be tightly controlled by the physiological state of *P. putida*. In fast growing bacteria, the expression of Fis is favoured, and the expression of RpoS is down-regulated [38], [39]. When the growth rate of bacteria decelerates and they enter stationary phase, the expressions of RpoS and Fis will be reversed [38], [39]. Waite *et al.* (2006) compared gene expression in planktonic and sessile *Pseudomonas aeruginosa* PAO1 by microarray analysis. As expected, they found that RpoS and Fis are oppositely regulated during biofilm development [40]. The level of *fis* mRNA was highest in the developing biofilm and slightly decreased in mature biofilm. Contrary to *fis*, the level of *rpoS* mRNA is highest in mature biofilm [40]. Considering the overlapping position of the *lapF* –10 element and Fis binding site Fis-F2, it is likely that Fis could repress transcription from the *lapF* promoter by impeding RNA polymerase binding. Therefore, it is more plausible that LapF expression is regulated by two competing regulators: Fis and RpoS. In logarithmically growing *P. putida* or at the beginning of biofilm development, LapF is not expressed or weakly expressed due to repression of Fis and the deficiency of activator RpoS. During growth speed deceleration or biofilm development, RpoS level increases and Fis level decreases, allowing LapF to be expressed. Thereby, the overexpression of Fis in stationary phase can unbalance the ratio of regulators leading to the Fis-induced transcriptional repression of *lapF* (Fig. 3) [8].

Summarizing the data presented in this report, we can conclude that Fis represses transcription from *lapF* promoter via the Fis-binding site Fis-F2. We identified a Fis binding site Fis-F2 *in vitro* by DNase I footprint and by using a β-galactosidase assay were able to show that Fis overexpression represses transcription from the *lapF* promoter region. Fis binding is weaker to the mutated Fis-F2-mut site and therefore Fis overexpression does not repress transcription from the *lapF* promoter. This confirms that Fis directly represses the *lapF* promoter.
